# Effects of Vegetable and Fruit Juicing on Gut and Oral Microbiome Composition

**DOI:** 10.3390/nu17030458

**Published:** 2025-01-27

**Authors:** Maria Luisa Savo Sardaro, Veronika Grote, Jennifer Baik, Marco Atallah, Katherine Ryan Amato, Melinda Ring

**Affiliations:** 1 Department of Anthropology, Northwestern University, Evanston, IL 60208, USA; jenniferbaik2024@u.northwestern.edu (J.B.); katherine.amato@northwestern.edu (K.R.A.); 2 Department for the Promotion of Human Sciences and Quality of Life, San Raffaele University, 00166 Rome, Italy; 3 Osher Center for Integrative Health, Northwestern University, Chicago, IL 60611, USA; veronikagrote2022@u.northwestern.edu; 4 Department of Food Science and Human Nutrition, University of Illinois Urbana-Champaign, Urbana, IL 61801, USA; marcoa3@illinois.edu

**Keywords:** diet recommendations, fruit juicing, gut microbiome, juice fasts, oral microbiome

## Abstract

Background: In recent years, juicing has often been promoted as a convenient way to increase fruit and vegetable intake, with juice-only diets marketed for digestive cleansing and overall health improvement. However, juicing removes most insoluble fiber, which may diminish the health benefits of whole fruits and vegetables. Lower fiber intake can alter the microbiota, affecting metabolism, immunity, and mental health, though little is known about juicing’s specific effects on the microbiota. This study addresses this gap by exploring how juicing impacts gut and oral microbiome composition in an intervention study. Methods: Fourteen participants followed one of three diets—exclusive juice, juice plus food, or plant-based food—for three days. Microbiota samples (stool, saliva, and inner cheek swabs) were collected at baseline, after a pre-intervention elimination diet, immediately after juice intervention, and 14 days after intervention. Moreover, 16S rRNA gene amplicon sequencing was used to analyze microbiota taxonomic composition. Results: The saliva microbiome differed significantly in response to the elimination diet (unweighted UniFrac: F = 1.72, R = 0.06, *p* < 0.005; weighted UniFrac: F = 7.62, R = 0.23, *p*-value = 0.0025) with a significant reduction in Firmicutes (*p* = 0.004) and a significant increase in Proteobacteria (*p* = 0.005). The juice intervention diets were also associated with changes in the saliva and cheek microbiota, particularly in the relative abundances of pro-inflammatory bacterial families, potentially due to the high sugar and low fiber intake of the juice-related products. Although no significant shifts in overall gut microbiota composition were observed, with either the elimination diet or the juice intervention diets, bacterial taxa associated with gut permeability, inflammation, and cognitive decline increased in relative abundance. Conclusions: These findings suggest that short-term juice consumption may negatively affect the microbiota.

## 1. Introduction

The global increase in chronic diseases in the last decades, particularly in industrialized populations characterized by a diet low in fiber and high in sugars and fats, highlights the importance of understanding the link between dietary habits and chronic diseases (CDs) [1,2]. Evidence-based dietary recommendations, informed by randomized trials, have explored causal relationships between specific dietary factors (e.g., processed food and trans-fat) and CDs such as heart disease, diabetes, and colorectal cancer [2,3,4,5,6,7,8,9,10]. Results have demonstrated that although the impact of individual dietary factors varies across countries, the non-optimal intake of three dietary factors (whole grains, fruits, and sodium) accounts for more than 50% of deaths and 66% of DALYs (disease-specific deaths and disability-adjusted life-years) attributable to diet [5]. These findings have been widely used to inform national and international dietary guidelines aimed at preventing CDs [11,12]. Within this context, a balanced diet that contains whole foods, especially fruits and vegetables, has been shown to have numerous health benefits [13,14,15]. Some fruits and vegetables tend to have a low glycemic load, resulting in smaller postprandial glucose spikes promoting satiety and weight maintenance [16,17]. In addition, fruits and vegetables are crucial sources of phenolic compounds, which may influence insulin sensitivity [18] and the anabolic state of adipose tissue [19]. Polyphenols are generally broken down in the colon, where they impact gut bacterial diversity with antioxidant, immunomodulatory, and antimicrobial properties [20,21,22,23]. The literature also highlights the health benefits of fruit and vegetable fibers [24,25,26,27] in the increase in satiety, reducing the total energy intake and preventing weight gain. Fibers serve as an important source of energy for the gut bacteria in the colon, influencing short-chain fatty (SCFA) production [24,28,29], which has a range of impacts on metabolism, immunity, and health.

In recognition of these health benefits, the 2020–2025 American Dietary Guidelines advise adults to consume 1.5–2 cups of fruits and 2–3 cups of vegetables per day [30]. However, meeting these dietary recommendations can be challenging, and strategies for ‘sneaking’ servings of fruits and vegetables, such as juicing, are receiving increasing attention. Moreover, there is growing public interest in the practice of “juice fasts” or “juice cleanses”, defined as short-term dietary practices during which a person voluntarily consumes only fruit, vegetable, or other plant juices; their extracts; or fruit teas [31]. The 2024 reports highlighted that 26% of consumers have tried a juice cleanse or detox program for perceived benefits like detoxification, weight loss, and improved digestion, and the market is growing accordingly [32].

Studies have demonstrated that juicing is a viable way to attain the nutritional benefits of fruits and vegetables, improving levels of beta-carotene, vitamin C, and vitamin E [33]. For example, recent evidence reviews concluded that 100% fruit and vegetable juices appear to have a beneficial or neutral effect on health [34] and cardioprotective effects, including lowering blood pressure and improving lipid profiles [35]. Nevertheless, the extraction process of juicing may negatively affect the health benefits of whole fruits and vegetables by altering properties such as fiber content [36]. These changes may influence how fruits and vegetables interact with consumer health via multiple pathways, including the gut microbiota. However, while previous studies have explored the association between the gut microbiota and fruit and vegetable consumption, few have investigated the association with juicing [20,37,38], which provides a unique context in which much of the insoluble fiber of fruits and vegetables has been extracted [36]. Also, depending on the fruits and vegetables used to make juice, sugar levels, total carbohydrates, and glycemic load can vary [39,40].

Even less is known about juicing’s impact on oral microbiota [20]. While much of the literature focuses on the gut, the oral cavity is the first to be exposed to exogenous factors, and there has been extensive evidence of microbial transmission from the mouth to the gut [41]. Additionally, inflammation in the oral cavity is associated with systemic inflammation and cardiovascular disease [42,43,44], insulin resistance [45], and complications in type 1 and type 2 diabetes [46,47]. The oral microbiome directly interfaces with host inflammation [48,49]. For instance, in periodontal disease, *Porphyromonas* species have been reported to induce the activation of the innate immune system [50], contributing to the development of rheumatoid arthritis [50], celiac disease [51], Alzheimer’s disease [52], Crohn’s disease [53], and cardiometabolic disease [54].

To address these gaps and advance our understanding of the impact of juicing on the gut and oral microbiota, we conducted a three-week dietary intervention study comparing two juice-based diets to a plant-based, whole-food diet. Longitudinal data of bacterial diversity and composition were performed on fecal and oral samples, including saliva and internal cheek swabs. We hypothesized that all diets would influence the oral and gut microbiome but that patterns would differ between body sites and that juice-based diets would increase bacteria associated with simple sugar metabolism, with greater changes in the oral microbiome compared to the gut, with a null hypothesis being the absence of variation. Our results contribute to the growing understanding of food processing effects on diet–microbiome–host interactions, with implications for refining dietary recommendations and food production practices.

## 2. Materials and Methods

### 2.1. Recruitment and Selection of Participants

Participants were recruited on the Northwestern University Evanston and Chicago campuses via flyers. We assessed 143 participants for eligibility using an online screening questionnaire and follow-up screening call between May 2018 and April 2019. The primary inclusion criteria were being between 18 and 35 years old, having a BMI between 18.5 and 30 kg/m^2^, and being considered healthy by the investigator based on medical history and completion of the screening questionnaire. Participants were excluded if they had a history of cardiovascular disease; daily use of any prescription or non-prescription medication that has a high likelihood of impacting systemic inflammation, blood sugar control, or the human microbiome; history of food allergy with hypersensitivity to any of the components of the juice or diet; or any diagnosis of allergic rhinitis, eczema, asthma, or inflammatory bowel disease. A total of 25 participants consented, and 23 participants completed the study activities. Moreover, 14 participants (7 females and 7 males) were used for the full analysis, with an average age of 22.7 years (SD = 2.8). All study participants provided written informed consent. The study was approved by the Northwestern University Institutional Review Board Human Subjects Committee (STU00206611) (Table S1, Supplementary Material).

### 2.2. Diet Intervention

Participants were randomized using a random number generator in Excel to follow one of three different diets for three days: (a) a typical “juice fast” of 800–900 Kcal exclusive cold pressed juice provided (Table S2, Supplementary Material) with 2 males and 3 females, (b) cold pressed juice plus regular diet ad libitum with 2 males and 2 females, (c) 800–900 Kcal plant-based, whole-food diet provided (Table S3, Supplementary Materials) with 3 males and 2 females. At Time Point 1, following the instructions typically provided as part of a juice fast, participants were asked to consume a three-day elimination diet consisting of organic fresh fruits, vegetables, gluten-free whole grains, eggs, and 8 glasses of water a day. They were advised to avoid or eliminate alcohol, caffeine, sugar, processed foods, dairy, red meat, and gluten (e.g., wheat, rye, barley, spelt). After three days, participants were asked to consume one of the three intervention diets listed above. Participants then followed their assigned 3-day reintroduction diet, after which they returned to their normal diets.

### 2.3. Specimen Collection

Fecal, saliva, and cheek samples were collected at baseline (Time Point 1), after the elimination diet (pre-intervention, Day 4, Time Point 2), immediately following the diet intervention (post-intervention, Day 7, Time Point 3), and 14 days after the intervention (Day 21, Time Point 4). Stool samples were collected using moistened wipes placed in collection plastic bags. Saliva samples were collected using the passive drooling method with the SpeciMAX™ Saliva Collection Kit (Fisher Scientific, Austin, TX, USA). Internal cheek samples were collected using the DNA Test Saliva Swab Collection Kit (DC Diagnocine Precision, Totowa, NJ, USA). All samples were kept in participants’ home freezers (−20 °C) wrapped in ice packs until they were transferred to the Amato Lab on ice packs and stored at −80 °C. The samples were collected from 14 individuals, yielding 56 saliva samples (an average of 4 samples per person), 54 cheek samples (an average of 3.85 samples per person), 43 fecal samples (an average of 3 samples per person). Some participants did not provide all four samples.

### 2.4. Sample Processing for 16S rRNA Gene Amplicon Sequencing

We extracted DNA from all samples using the commercial DNeasy Powersoil Pro Kit (Qiagen, Germantown, MD, USA) with modifications. Briefly, after adding solution C1, samples were incubated at 65 °C for 15 min before vortexing for 10 min. Additionally, we warmed solution C6 at 65 C before adding. A two-step PCR was used to amplify the V4–V5 region of the 16S rRNA gene, utilizing the 515 forward and 926 reverse Earth Microbiome Project primers (www.earthmicrobiome.org), as described previously [55,56]. PCR products were purified and normalized using SequalPrep Normalization. Sequencing of barcoded amplicons was performed on an Illumina MiSeq V4 platform by the Rush Genomics and Microbiome core facility with a depth of at least 20,000 sequences per sample. Negative controls from DNA extractions and PCRs were included in the initial data set and used as negative controls for contamination.

Raw sequences were demultiplexed and quality filtered using the q2-demux plugin, followed by denoising with DADA2 [57] (via q2-dada2) in the QIIME2 2020.6 pipeline [58]. Sequences from mitochondria and chloroplasts were removed. Amplicon sequence variants (ASVs) were identified with a learned sequencing error correction model (DADA2 method). The dataset contained an average of 11,695 reads per sample for fecal samples (range: 15,790–4095), an average of 7898 reads per sample for saliva (range: 22,427–6630), and an average of 8113 reads per sample for cheek samples (range: 22,641–6414). Alpha-diversity metrics (Shannon diversity, Observed ASVs, Faith’s Phylogenetic Diversity [59]), and beta diversity metrics (weighted UniFrac [60], unweighted UniFrac [61], Jaccard, Bray–Curtis [62,63]) were estimated using the q2-diversity plug-in after samples were rarefied to 7000 reads per sample. Taxonomy was assigned to ASVs using the q2-feature-classifier [64] against Greengenes v.gg-13-8-99-nb-classifier [65]. The taxonomic composition of the negative controls was compared to the true (non-control) samples, and there was no indication of contamination. Based on these verifications, the negative controls were removed from the dataset that was used in subsequent statistical analyses.

### 2.5. Statistical Analysis

Once processed, we used sequence data to compare richness, diversity, and microbial community composition among samples. Stool, saliva, and internal cheek samples were analyzed separately. We first tested the effect of the elimination diet by comparing baseline and pre-intervention time points in all individuals together. We then tested the effect of the intervention diet by comparing the pre-intervention, immediate post-intervention, and 14-day post-intervention time points for each diet separately. The differences between diets were evaluated by comparing the juice intervention diet to the juice plus food ad libitum and plant-based diets at the two post-intervention time points.

All statistical analyses were performed in R (version 3.4.0). The QIIME outputs and associated metadata were imported into R as a *phyloseq* object using QIIME2R package (version 0.99.6) [66]. For each sample type, differences in microbial richness and alpha diversity across diets (Shannon, Observed ASVs) were visualized by using the *phyloseq* package [67], *ggplot2* [68], and *ggsignif* [69] packages in R and tested using Wilcoxon rank sum and Kruskal–Wallis tests (*stats* package; R software (version 3.4.0). Differences in the microbial composition of the oral cavity and gut in response to the elimination diet and intervention diets were visualized using non-metric multi-dimensional scaling (NMDS) with unweighted and weighted UniFrac distance matrices using the vegan package [70]. We tested the observed patterns using permutational analysis of variance (PERMANOVA) with the adonis2 function in the vegan package. We visualized the differences in the relative abundances of bacterial phyla across sample groups using bar plots in ggplot2. We also generated differential abundance heatmaps based on the centered log-ratio (CLR) values of the initial dataset for bacterial families with relative abundances higher than 0.2% using the Complex Heatmap [71,72] package with Aitchison distance dendrograms [73,74]. A heatmap of the relative abundances of bacterial species at baseline and Time Point 1 was generated using *phyloseq* [67], the qiime2R *taxa_heatmap* function [66], and ggplot2, with the criterion that the taxa exceed 0.1% relative abundance in over 90% of the samples (prevalence ≥ 0.9). The relationship between the relative abundances of bacterial phyla in the oral and gut microbiota and diet treatment were tested using a fixed linear regression model with the *lme* function in R. We also tested for differential abundance of bacterial phyla and families using Analysis of Composition of Microbiomes (ANCOM-BC) [75,76]. The influence of sex was assessed using pairwise comparisons analysis within the PairwiseAdonis R package (v0.4) [77]. This analysis considered the three body sites and the four time points of the diet interventions. However, since no significant differences were observed (Table S4), both sexes were grouped for the subsequent analysis. Given the limited sample size, we present both significant statistical results and noteworthy non-significant trends in our heatmaps.

## 3. Results

### 3.1. Effects of the Elimination Diet: Baseline Versus Pre-Intervention Time Points

#### 3.1.1. Oral Microbiome

**Figure 1:**
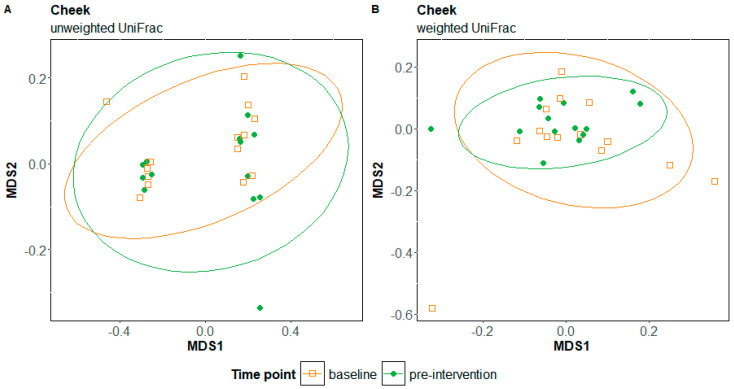
Non-metric multidimensional scaling (NMDS) plots of unweighted UniFrac (**A**) and weighted UniFrac distances (**B**) for cheek samples at baseline and pre-intervention time points, in response to the elimination diet; MDS: metric multidimensional scaling.

**Figure 2:**
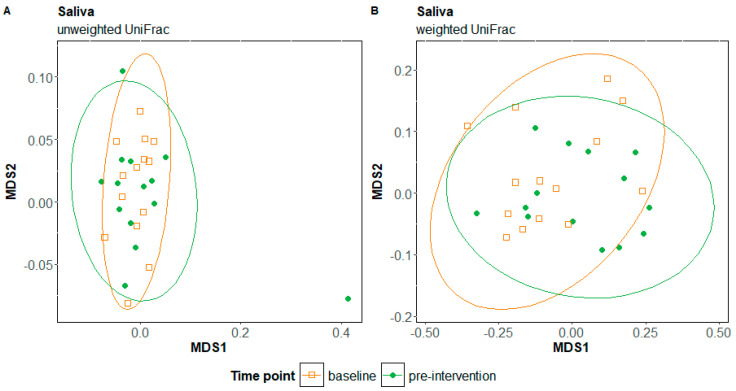
Non-metric multidimensional scaling (NMDS) plots of unweighted UniFrac (**A**) and weighted UniFrac distances (**B**) for saliva samples at baseline and pre-intervention time points, in response to the elimination diet; MDS: metric multidimensional scaling.

**Figure 3:**
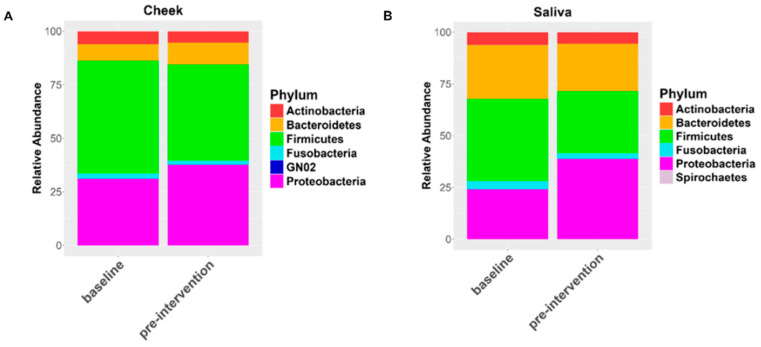
Relative abundances of bacterial phyla in cheek samples (**A**) and saliva samples (**B**) at baseline at pre-intervention time points in response to the elimination diet.

Microbial diversity did not differ in response to the elimination diet for either the internal cheek microbiome or the saliva microbiome (Figures S1 and S2). The composition of the internal cheek microbiome also did not differ significantly in response to the elimination diet (unweighted UniFrac: F = 1.39, R = 0.51, *p* = 0.12; weighted UniFrac: F = 0.90, R = 0.003, *p* = 0.5; Figure 1A,B). In contrast, the composition of the saliva microbiome differed significantly in response to the elimination diet (unweighted UniFrac: F = 1.72, R = 0.06, *p* < 0.005; weighted UniFrac: F = 7.62, R = 0.23, *p*-value = 0.0025; Figure 2A,B). The regression analysis at the phylum level showed a slight but significant decrease in the relative abundance of Firmicutes (*p* = 0.01) and a slight significant increase in the relative abundance of Proteobacteria in cheek samples (*p* = 0.05) (Figure 3A). We observed the same patterns with a greater magnitude in the saliva microbiome (reduction in Firmicutes: *p* = 0.004; increase in Proteobacteria: *p* = 0.005 (Figure 3B). The increased relative abundance of Proteobacteria in the cheek and saliva is mainly driven by variation in the relative abundance of *Neisseria* sp. and *Haemophilus parainfluenzae*, whereas the reduced relative abundance of Firmicutes is driven by a logarithmic reduction in the relative abundance of *Lachnospiraceae* sp. (Figures S3 and S4). ANCOM generates W values between 2600 and 2800 and a positive CLR of 10 for these taxa.

#### 3.1.2. Gut Microbiome

**Figure 4:**
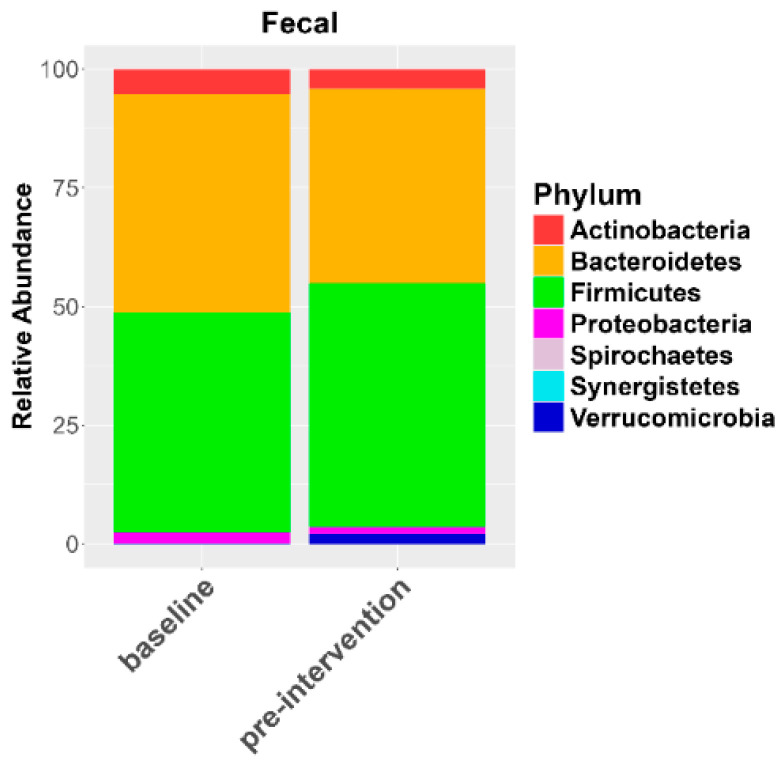
Relative abundance of bacterial phyla in fecal samples in response to the elimination diet at baseline and pre-intervention time points.

**Figure 5:**
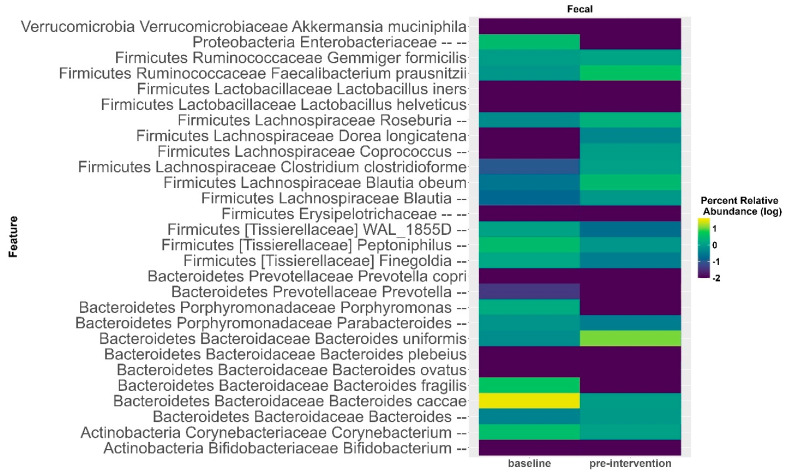
Relative abundance heatmap of genus and bacterial species in fecal samples in response to elimination diet, the baseline and pre-intervention time pointsare reported as log10 of percent abundance.

Similar to what was observed in the oral microbiome, there were no significant differences in gut microbial diversity in response to the elimination diet (Figure S6, Shannon diversity: F_1,15_ = 1.579, *p* = 0.228; Faith’s PD: F_1,15_ = 1.332, *p* = 0.26). In contrast to the oral microbiome, the composition of the gut microbiota also did not differ significantly in response to the elimination diet (Figure S5A,B; unweighted UniFrac: F = 0.96, R = 0.06, *p* = 0.53; weighted UniFrac: F = 0.82, R = 0.05, *p* = 0.55). However, regression identified a slight significant increase in the relative abundance of Firmicutes (*p* = 0.017) in response to the elimination diet (Figure 4). Accordingly, there is a trend for increased relative abundances of Firmicutes species in response to the elimination diet (Figure 5). The heatmap and ANCOM-BC results showed a 0.15 Log Fold (LFC) increase in *Faecalibacterium prausnitzii* (*q*-value of 1), a 0.66 LFC increase in *Blautia obeum* (*q*-value of 1), a 0.57 LFC in *Blautia producta* (*q*-value of 1), a 0.46 LFC in *Dorea longicatena* (*q*-value of 1), and a slight increase in *Roseburia* spp. and in *Coprococcus* spp. There was also an increase in non-Firmicutes taxa, such as an increase in 0.36 LFC of *Bacteroides uniformis* (*q*-value of 1). We also observed a reduction in the relative abundances of *Enterobacteriaceae* spp., *Bacteroides fragilis* (−0.0005 LFC, *q*-value of 1) and *Bacteroides caccae* (−1.25 LFC, *q*-value of 1), and *Corynebacterium aurimucosum* (LFC −1.86, *q*-value of 0.7).

### 3.2. Effects of the Intervention Diets: Pre-Intervention Versus Immediate Post-Intervention and 14-Day Post-Intervention

#### 3.2.1. Oral Microbiome

**Figure 6:**
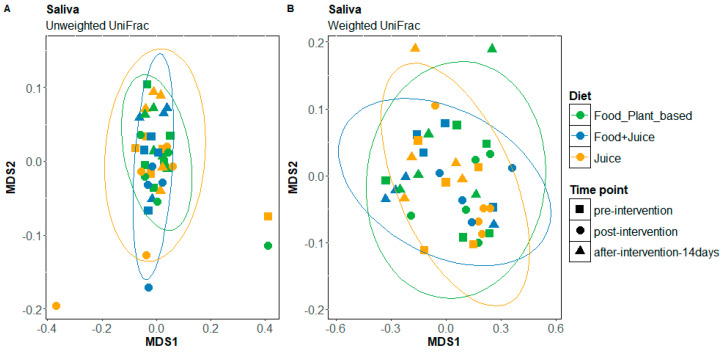
Non-metric multidimensional scaling (NMDS) plots with the unweighted Unifrac (**A**) and weighted Unifrac distances (**B**) of saliva samples at pre-intervention, post-intervention, and 14-day post-intervention time points for the three diet types.

There were no differences in microbial diversity in response to any of the three intervention diets for cheek or saliva samples (*p* > 0.01) (Figures S7 and S8). There were also no differences in cheek microbiome composition in response to the intervention diets (unweighted UniFrac: F = 0.86, R = 0.044, *p* = 0.58 and weighted UniFrac: F = 1.23, R = 0.17, *p* = 0.18) (Figure S9A,B), but there were differences for the saliva microbiome (unweighted UniFrac: F= 1.27, R = 0.18, *p* = 0.0008, weighted UniFrac: F = 4.06, R = 0.42, *p* = 0.0002 (Figure 6A,B). The effects of the exclusive juice diet were driven by differences in the presence/absence of bacterial taxa after three days of intervention (unweighted UniFrac: F = 1.98, R = 0.19, *p* = 0.02) as well as the relative abundances of bacterial taxa after 14 days of treatment (weighted UniFrac: F = 9.71, R = 0.36, *p* = 0.019). The effects of the juice plus food diet on saliva microbiome composition were driven by differences in the presence/absence of bacterial taxa after three days of diet intervention (unweighted UniFrac: F = 1.65, R = 0.093, *p* = 0.0005) and in both the presence/absence of bacterial taxa (unweighted UniFrac: F = 2.32, R = 0.126, *p* = 0.0041) as well as the relative abundances of bacterial taxa (weighted UniFrac: F = 15.2, R = 0.48, *p* = 0.016) 14 days after intervention. For the plant-based diet, only the relative abundances of taxa showed significant differences after three days of treatment (weighted Unifrac: F = 10.7, R = 0.38, *p* = 0.001).

**Figure 7:**
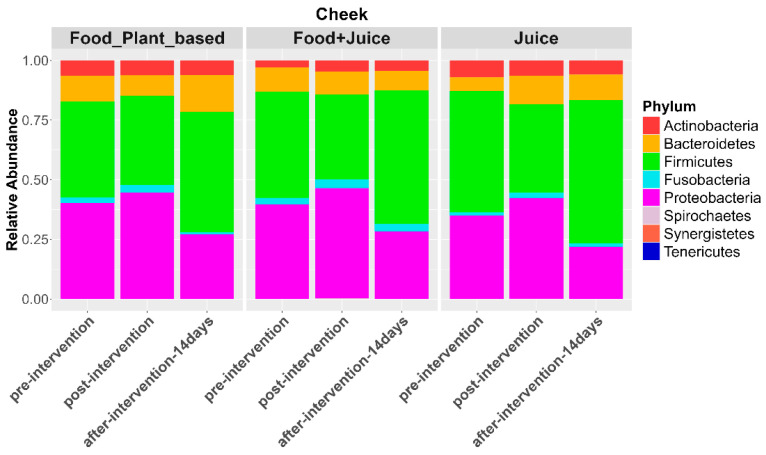
Relative abundance of bacterial phyla in cheek samples at pre-intervention, post-intervention, 14 days post intervention for the three diet types.

**Figure 8:**
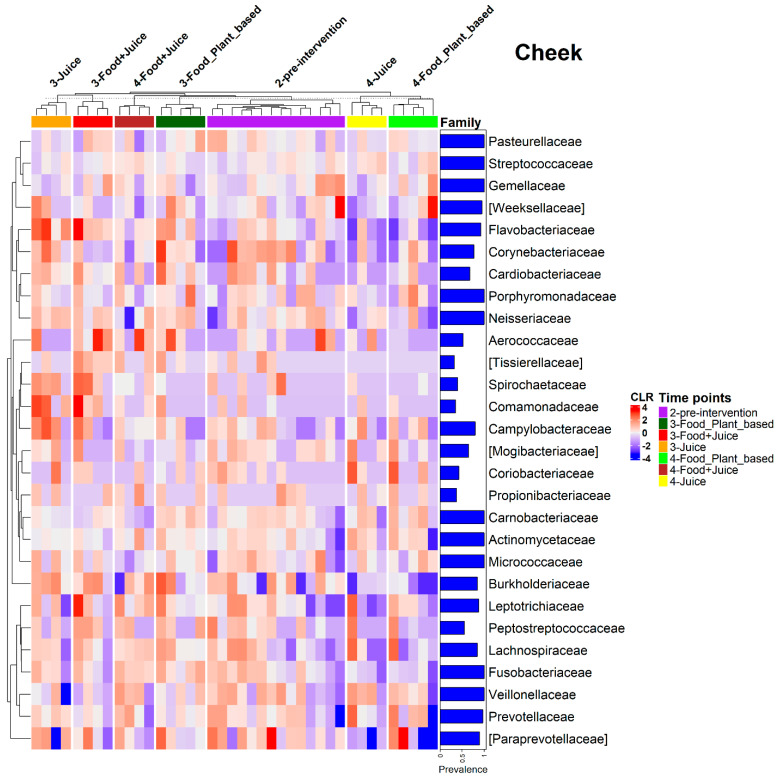
Relative abundance heatmap on the centered log-ratio (CLR) transformed values of bacterial families with prevalence higher than 20% in cheek samples in the three diet types at pre-intervention (2-pre-intervention), post-intervention (3-Food_Plant_based, 3-Food+Juice, 3-Juice), 14 days post intervention (4-Food_Plant_based, 4-Food+Juice, 4-Juice) time points.

Irrespective of changes in overall composition, both the internal cheek microbiome and saliva microbiome exhibited shifts in the relative abundances of individual taxa in response to diet interventions (Figure 7). These changes were most similar between the juice and juice plus food diets. For the cheek microbiome, the exclusive juice diet resulted in a significant increase in the relative abundance of Proteobacteria (*p* = 0.005) and a reduction in the relative abundance of Firmicutes (*p* = 0.001). The juice plus food diet resulted in a significant reduction in the relative abundance of Firmicutes (*p* < 0.005). The plant-based diet was associated with a reduction in the relative abundances of Bacteroidetes (*p* = 0.03) and Firmicutes (*p* = 0.04) and an increase in the relative abundances of Fusobacteria (*p* = 0.02) and Proteobacteria (*p* = 0.004; Figure 7). ANCOM-BC analysis indicated a range of families driving these patterns. For example, for the juice diet, there was a 0.84 Log Fold (LFC) increase in *Comamonadaceae,* a 1.46 LFC increase in *Aerococcaceae* (*q*-value of 0.11), and a 0.49 LFC increase in *Flavobacteriaceae.* For the food plus juice diet, there was a 0.57 LFC increase in *Comamonadaceae,* a 1.3 LFC (*q*-value of 0.53) in *Aerococcaceae,* and a 0.64 LFC in *Flavobacteriaceae* (*q*-value of 0.018). Additionally, *Burkholderiaceae*, *Fusobacteriaceae*, *Neisseriaceae*, and *Cardiobacteriaceae* showed increased relative abundances across all three dietary interventions, whereas *Veillonellaceae* showed decreased relative abundances. We also observed trends in other families, such as increased *Spirochaetaceae* in response to the exclusive juice and juice plus food diets, as well as increased *Tissiellaceae* in the juice plus food diet (Figure 8).

**Figure 9:**
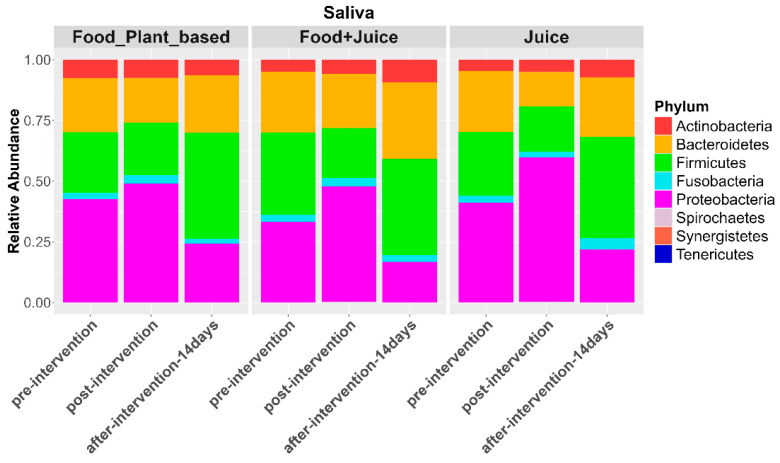
Relative abundance of bacterial phyla in saliva samples at pre-intervention, post-intervention, 14 days post intervention for the three diet types.

For the saliva microbiome, patterns were similar to those observed in the cheek. The exclusive juice diet exhibited a strong increase in the relative abundance of Proteobacteria (*p* = 0.00003) and a decrease in the relative abundance of Firmicutes (*p* = 0.0001; Figure 9). Similarly, the juice plus food diet was associated with an increase in the relative abundance of Proteobacteria (*p* = 0.03) and a decrease in the relative abundances of Firmicutes (*p* = 0.0004) and Actinobacteria (*p* = 0.0001). The plant-based diet showed a slight increase in the relative abundance of Proteobacteria (*p* = 0.01) and a reduction in the relative abundance of Firmicutes (*p* = 0.0008) over time.

**Figure 10:**
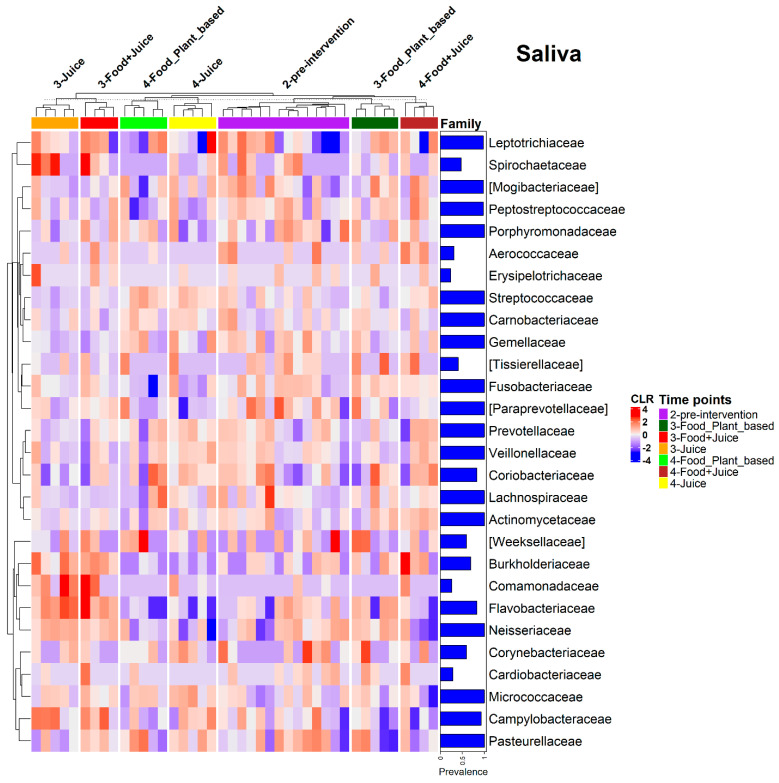
Relative abundance heatmap on the centered log-ratio (CLR) transformed values of bacterial families with prevalence higher than 20% in saliva samples in the three diet types at pre-intervention (2-pre-intervention), post-intervention (3-Food_Plant_based, 3-Food+Juice, 3-Juice), 14 days post intervention (4-Food_Plant_based, 4-Food+Juice, 4-Juice) time points.

Patterns in bacterial families contributing to observed changes in bacterial phyla in the saliva were similar to those observed in the cheek samples. ANCOM-BC analysis demonstrated an increased relative abundance of *Flavobacteriaceae* (1.52 LFC, *q*-value *=* 0.0009 post-intervention), *Spirochaetaceae* (1.94 LFC, *q*-value *=* 0.0018), *Campylobacteraceae* (0.11 LFC, *q*-value = 1), and *Burkholderiaceae* 0.64 LFC (*q*-value = 1) in the exclusive juice diet. We also observed an increased relative abundance of *Flavobacteriaceae* (2.04 LFC, *q*-value *=* 0.0009 post-intervention), *Spirochaetaceae* (1.58 LFC, *q*-value = 0.06), *Campylobacteraceae* (0.46 LFC, *q*-value = 1), and *Burkholderiaceae* (1.04 LFC, *q*-value = 0.48) in the exclusive juice diet. Additionally, *Fusobacteriaceae* and Neisseriaceae showed increased relative abundances across all three diets, whereas *Streptococcaceae* showed a decreased relative abundance. *Prevotellaceae*, *Veillonellaceae*, *Coriobacteriaceae*, and *Lachnospiraceae* also had reduced relative abundances in the exclusive juice and juice *plus* food interventions. Finally, we observed trends in other families, such as increased *Spirochaetaceae* in response to the exclusive juice and juice plus food diets, as well as increased *Tissiellaceae* in the food plus juice diet (Figure 10).

#### 3.2.2. Gut Microbiome

**Figure 11:**
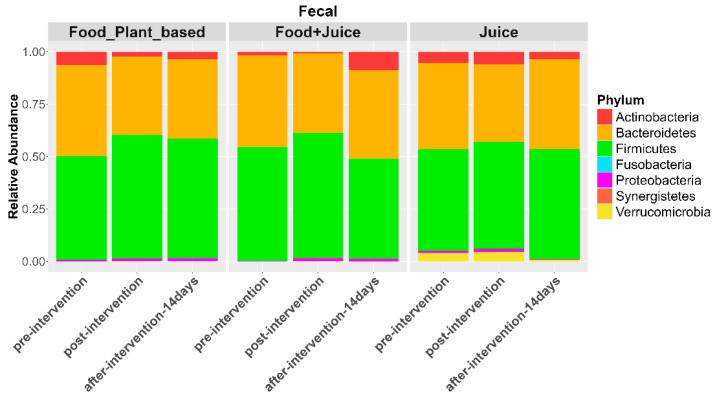
Relative abundance of bacterial phyla in fecal samples at pre-intervention, post-intervention, 14 days post intervention for the three diet types.

There were no significant differences in gut microbial diversity (Figure S10) or composition (Figure S11) in response to any of the diet interventions (*p* > 0.01). However, there was more inter-individual variation in gut microbiome composition over time among participants who consumed the plant-based and juice diets compared to the juice plus food group (Figure S11). Additionally, the regression model did not show significant differences in the relative abundances of bacterial phylum ratio in response to any diet (Figure 11).

**Figure 12:**
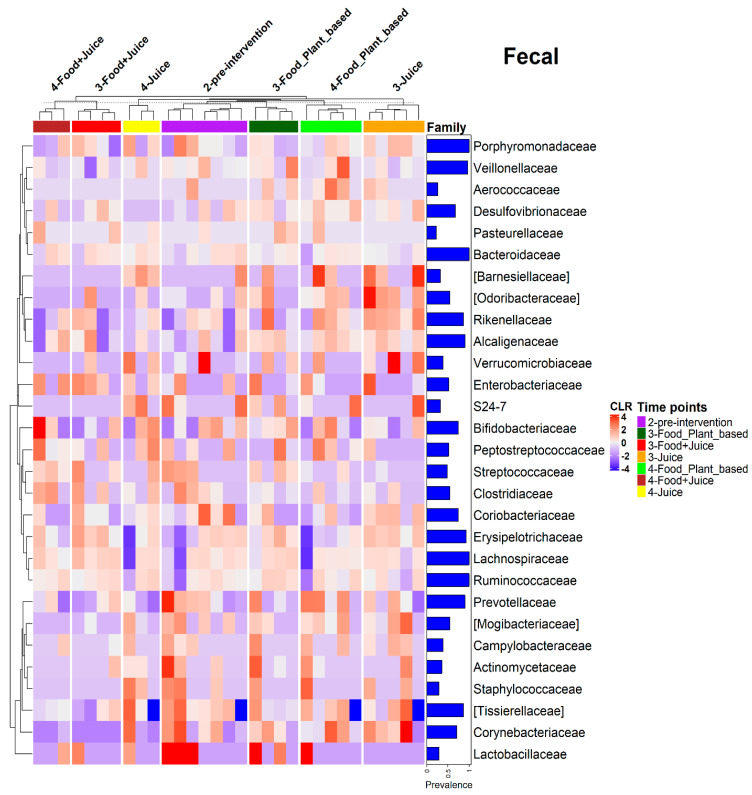
Heatmap based on the centered log-ratio (CLR) transformed values of bacterial families with prevalence higher than 20% in fecal samples in the three diets intervention at pre-intervention (2-pre-intervention), post-intervention (3-Food_Plant_based, 3-Food+Juice, 3-Juice), 14 days post intervention (4-Food_Plant_based, 4-Food+Juice, 4-Juice) time points.

ANCOM-BC also did not demonstrate any significant changes in the relative abundances of bacterial families. However, there was a trend for an increased relative abundance of *Porphyromonadaceae* (2.5 LFC *q*-value = 1), *Rikenellaceae* (1.43 LFC), *Coriobacteriaceae* (1.14 LFC), *Alcaligenaceae* (0.63 LFC), and *Erysipelotrichacaeae* (0.88 LFC) in the exclusive juice diet (Figure 12). There was also a trend for increased relative abundances of *Porphyromonadaceae* (1.76 LFC, *q*-value = 0.43) and *Erysipelotrichaceae* (0.89 LFC) in the juice *plus* food diet (Figure 12). We also observed trends for increased *Odoribacteraceae*, *Rikenellaceae*, *Tissierellaceae*, and *Corynebacteriaceae* in the exclusive juice diet and increased *Coriobacteriaceae* and *Enterobacteriaceae* in the juice plus food diet (Figure 12).

Considering our hypothesis that all diets would be associated with changes in the oral and gut microbiome with different patterns between body sites and a stronger impact by the juice-based diets, we observed specific bacterial patterns that were body site-specific. We also noted variation associated with the three different diet interventions, with greater bacterial variation observed in both body sites with the juice intervention but with a higher responsiveness in the oral microbiota compared to the gut microbiota, which showed limited bacterial variation after three days of diet intervention (Figure S12). Saliva exhibited the highest variation in bacterial species and abundance, as confirmed by the previously discussed beta diversity and the linear regression analysis, as also evident in Figure S12B. Moreover, the overall comparison of the baseline, pre-intervention, post-intervention, and 14-day-after-intervention time points for the three diet interventions (Figure S12) shows a general trend toward returning to the initial baseline composition.

## 4. Discussion

In this study, we aimed to improve our understanding of the impact of juicing on the gut and oral microbiome using a three-week dietary intervention study comparing two juice-based diets to a plant-based, whole-food diet. As hypothesized, these diets affected the oral and gut microbiome differently, with bigger effects on the oral gut microbiome. As predicted, particularly in the oral microbiome, juice-based diets were associated with increased relative abundances of bacterial taxa that process simple sugars. Our results contribute to our knowledge of how juicing affects health and should be considered in future studies and clinical interventions.

To begin with, we found that the saliva microbiome was highly sensitive to diet variation, with rapid and significant changes in response to both the elimination diet and the intervention diets. The patterns observed in both saliva and cheek samples were similar. However, the saliva microbiome was the most responsive. This dynamic is likely related to the presence of biofilms on the cheek that may be able to buffer the short-term diet shifts [78]. In contrast to the oral gut microbiome, the gut microbiome changed very little in response to any of the diet treatments. These results align with Henning et al. (2017), who did not find significant variation in the gut microbiome after a four-day intervention with a vegetable/fruit juice-based diet [20]. However, they did observe significant reductions in Firmicutes and Proteobacteria relative abundances and an increase in Bacteroidetes relative abundance, with a specific increase in *Bacteroides*, which is associated with fiber degradation. The distinct dynamics at the taxa level are most likely related to the different amounts of sugar and total carbohydrates in the diets provided in the two studies. For example, our exclusive juice diet had a higher content of sugar (mean of 13.1 g in the vegetable/fruit juice-based diet versus ~20.6 g of the exclusive juice diet) and total carbohydrates (mean of 18.8 g versus 38.6 g) despite having a similar amount of fiber (2.3 g versus 2.6 g). The increased abundance of Proteobacteria that we observed could be a result of its ability to take advantage of simple sugars rapidly and, therefore, grow quickly [79,80]. The increased relative abundances of Proteobacteria in the oral cavity were also evident in our plant-based whole food diet that contained a relatively high level of total carbohydrate (~31 g) and sugar (9.3 g) despite a higher amount of fiber (~6.5 g). As a result, beyond simply testing the effects of different diet categories on the microbiome and health, it is important to consider the actual nutritional composition of the diets.

In addition, these patterns highlight the importance of controlling the timing of sample collection in relation to food consumption in oral microbiome studies. Pulses of sugar from food may result in distinct microbiome dynamics at different time points after food consumption, given how immediately food interacts with the oral microbiome after it is consumed and how quickly it exits the oral cavity. This concern is less important for the gut microbiota studies given that foods pass through more stages of digestion before reaching the large intestine, and there is less temporal variability in the exposure of the gut environment to food. As a result, the gut is generally characterized by a more stable microbial community. Despite this trait, we did still observe some changes in response to diet, such as an increase in Firmicutes in all three diet interventions. These patterns could be correlated to a consistently higher level of fermentable carbohydrates in the intervention diets compared to participants’ normal diets and should be explored more in future studies.

Interestingly, one of the biggest diet effects we observed in this study was the elimination diet. Despite the potential health risks observed with the elimination diet in the oral microbiota with increased Proteobacteria, an inflammatory microbe [81,82], *Neisseria* sp. [83] and *Haemophilus parainfluenzae* [84,85] were both also detected at higher levels. These taxa are both commonly isolated from the mouth of healthy individuals and are not typically associated with oral disease, with *Neisseria* sp. also reported to have a possible physiological role in preventing colonization of potential pathogens [86]. Additionally, we observed a reduction in *Lachnospiraceae* sp. This taxon has been correlated with cognitive impairment [87], making the observed pattern potentially positive for health.

The gut microbial changes associated with the elimination diet generally appeared to be positive with regard to potential health benefits. These changes included an increase in *Faecalibacterium prausnitzii*, which produces butyrate [88,89], a crucial compound for maintaining healthy gut physiology and host wellbeing. Specifically, butyrate is a main energy source for the colonocytes with protective properties against colorectal cancer (CRC) and immune-inflammatory chronic diseases like inflammatory bowel diseases (IBD) and Crohn’s disease [90,91,92,93]. Butyrate has been shown to reduce intestinal mucosa inflammation by inhibiting NF-κB transcription factor activation [94], upregulating PPARγ [95], and inhibiting interferon gamma (IFN-γ;) [96]. Additionally, we observed increased relative abundances of members of the Lachnospiraceae family, such as *Blautia*, *Coprococcus*, *Dorea*, and *Roseburia*, that have been associated with beneficial effects on human health such as production of butyrate and other pathways involved in the control of gut inflammatory processes, atherosclerosis, maturation of the immune system [97,98,99,100], conversion of the primary bile acids in secondary bile acids [101], and antibiotic production in the human gastrointestinal tract linked to a reduction in pathogenic infections in children [102,103]. The *Blautia obeum* species we detected in response to the pre-intervention diet has also been characterized by its ability to produce a Novel Lantibiotic Nisin O antibiotic [102,103]. Positive health effects are also associated with *Bacteroides uniformis*, a fiber-degrading bacteria that promotes the digestion of complex fibers and is implicated in restoring the adaptive and innate immune cell balance that helps reverse immuno-metabolic dysfunction in obese mice [104]. We also observed an increase in the well-known probiotic *Bifidobacterium* spp. [105]. The pre-intervention diet also showed the possibility of controlling the growth of *Bacteroides fragilis*, a species involved in infections and identified also implicated in colorectal cancer [106,107,108], as well as *Bacteroidetes caccae*, which is involved in IBD and bloodstream infections [108,109], and the potential pathogen, *Enterococcus* spp. [110].

Diet interventions also affected the oral and gut microbiomes, though the impacts were smaller and tended to suggest negative health effects. For example, many of the taxa that increased in relative abundance in response to the juice and juice *plus* food diets have been identified as potential critical risk factors for their involvement in increasing inflammatory markers, colorectal cancer, cardiovascular disease, gingivitis, and periodontal disease. For example, in the oral microbiota, the reduction in Veillonellaceae could be important, as this family plays a role in producing nitrite by converting endogenous and exogenous sources of nitrate. Nitrite has an inhibitory effect on the growth of periodontal bacteria and can help reduce acid production from these strains [111,112,113], which affects caries formation. Even in the gut, where changes were less marked, we observed higher health concerns with the juice-exclusive diet due to an increase in the relative abundance of taxa correlated to proinflammatory activity. For example, the overrepresentation of taxa belonging to the *Porphyromonadaceae* and *Odoribacteraceae* families have been identified in an aging mouse study to induce memory deficits and increase anxious behavior with progression toward neurodegenerative disorder [114]. Also, the *Alcaligeneceae* family has been positively correlated with cognitive impairment [115]. The *Porphyromonadaceae*, *Rikenellaceae*, and *Coriobacteriaceae* families have also been found to be overrepresented in stressed mice induced into a depression-like state [116]. Finally, *Porphyromonadaceae* and *Odoribacteraceae* have been correlated with anxiety-like behavior and increased gut permeability and inflammatory markers in aged mice [117].

Despite these potential health concerns, it is important to note that, overall, the changes we found in both the oral and gut microbiota were somewhat small in magnitude. Given the extreme nature of some of the diet interventions, particularly the exclusive juice diet, this finding was unexpected. However, our intervention only lasted for three days. Given that many juice cleanses or diets that add juice may last for longer or are consumed periodically over time, we expect the potential cumulative negative effects may indeed be higher. Future studies should examine these dynamics in more detail. Additionally, the 14-day post-intervention period showed a trend toward the reestablishing of the pre-intervention microbiota composition, demonstrating that three days of diet intervention cannot be considered sufficient to significantly modulate the microbiota composition.

However, the small number of participants and the short duration of diet interventions limited our power to discriminate the species and strains involved and the reduced sample size has affected the overall accuracy of the findings and the ability to identify variations. For future studies related to dietary intervention recommendations, it is suggested to have a larger participant sample size and an extended time of intervention to observe a more stable variation in the gut and oral microbiota.

Our findings offer valuable insights for future food-health-related studies, highlighting the potential of saliva samples as a rapid tool to assess the short-term effects of specific ingredients or food products on oral microbiome composition. Meanwhile, cheek samples could serve to measure the lasting effects of dietary patterns, complementing the gut microbiome analyses.

## 5. Conclusions

This study highlights the critical health impacts of intervention diets, particularly an exclusive juice diet. Results showed changes in oral microbiota, particularly in the pro-inflammatory bacteria families. The gut microbiota also showed an increase in taxa associated with gut permeability, inflammation, and cognitive decline. These findings suggest that short-term juice consumption may negatively affect the microbiota, likely due to reduced fiber and the higher sugar and carbohydrate content, underscoring the need for further research on diet–microbiome–disease interactions, as juice products are also often considered a fruit substitute in children’s daily diets.

In contrast, the elimination diet showed beneficial effects on gut microbiota, increasing fiber-degrading and butyrate-producing bacteria that support gut inflammation control, immune balance, and resistance to pathogens, likely due to the higher dietary fiber content.

This study offers insights for redefining dietary recommendations and improving food production, prioritizing higher fiber products, reintegrating fiber into processed foods, and reducing sugar content to meet the demand for health-focused products.

## Data Availability

The raw sequence files are available at the NCBI Sequence Read Archive (https://www.ncbi.nlm.nih.gov/) under BioProject ID number PRJNA1215739.

## Supplementary Materials

The following are available online at https://www.mdpi.com/article/10.3390/nu17030458/s1, Figure S1: Observed ASVs and Shannon alpha diversity violin plots in cheek samples at baseline and pre-intervention time points in response to the elimination diet; Figure S2: Observed ASVs and Shannon alpha diversity violin plots in saliva samples at baseline and pre-intervention time points in response to the elimination diet; Figure S3: Heat map comparison of the relative taxa abundance in cheek samples from participants at baseline point and pre-intervention; Figure S4: Relative abundance heatmap of genus and bacterial species in saliva samples from baseline and pre-intervention intervention time points, in response to elimination diet, reported as log10 of percent abundance; Figure S5: Non-metric multidimensional scaling plots (MDS) with the Unweighted Unifrac (A) and Weighted Unifrac distance (B) on fecal samples at baseline and pre-intervention time point, in response to elimination diet; Figure S6: Observed ASVs and Shannon alpha diversity violin plots in fecal samples at baseline and pre-intervention time points, in response to the elimination diet; Figure S7: Shannon diversity violin plots in cheek samples at pre-intervention, post-intervention and 14 days post-intervention time points for the three diet types; Figure S8: Shannon diversity violin plots in saliva samples at pre-intervention, post-intervention and 14 days post-intervention time points for the three diet types; Figure S9: Multidimensional scaling plots (MDS) on Unweighted Unifrac (A) and Weighted Unifrac distance matrix (B) performed on cheek samples at pre-intervention, post-intervention and after-intervention-14days; Figure S10: Multidimensional scaling plots (MDS) on Unweighted Unifrac (A) and Weighted Unifrac distance matrix (B) performed on fecal samples at pre-intervention, post-intervention and after-intervention-14days; Figure S11: Shannon diversity violin plots in fecal samples at pre-intervention, post-intervention and 14 days postr-intervention time points for the three diet types; Figure S12: Relative abundance of bacterial phyla in (A) Cheek, (B) saliva, and (C) fecal samples at baseline (1-baseline), pre-intervention (2-pre-intervention), post-intervention (3-Food_Plant_based, 3-Food+Juice, 3-Juice), 14 days post intervention (4-Food_Plant_based, 4-Food+Juice, 4-Juice) time points. Table S1: Diet intervention summary; Table S2: Nutritional composition of the juices consumed as part of the exclusive juice diet and the juice plus food interventions; Table S3: List of the food products used for the plant-based, whole food intervention; Table S4: Pairwise Comparisons analysis based on sex factor and diet interventions using weighted UniFrac and unweighted UniFrac distances across the three body sites: cheek, saliva, and fecal samples.
