# Supplementary material for: Effects of Vegetable and Fruit Juicing on Gut and Oral Microbiome Composition
Source: Nutrients. 2025 Jan 27;17(3):458. doi: 10.3390/nu17030458 (PMC11820471; doi:10.3390/nu17030458)
Supplement: Supplementary file 1 [file nutrients-17-00458-s001.zip › nutrients-3415989-supplementary.pdf]

Supplementary Material

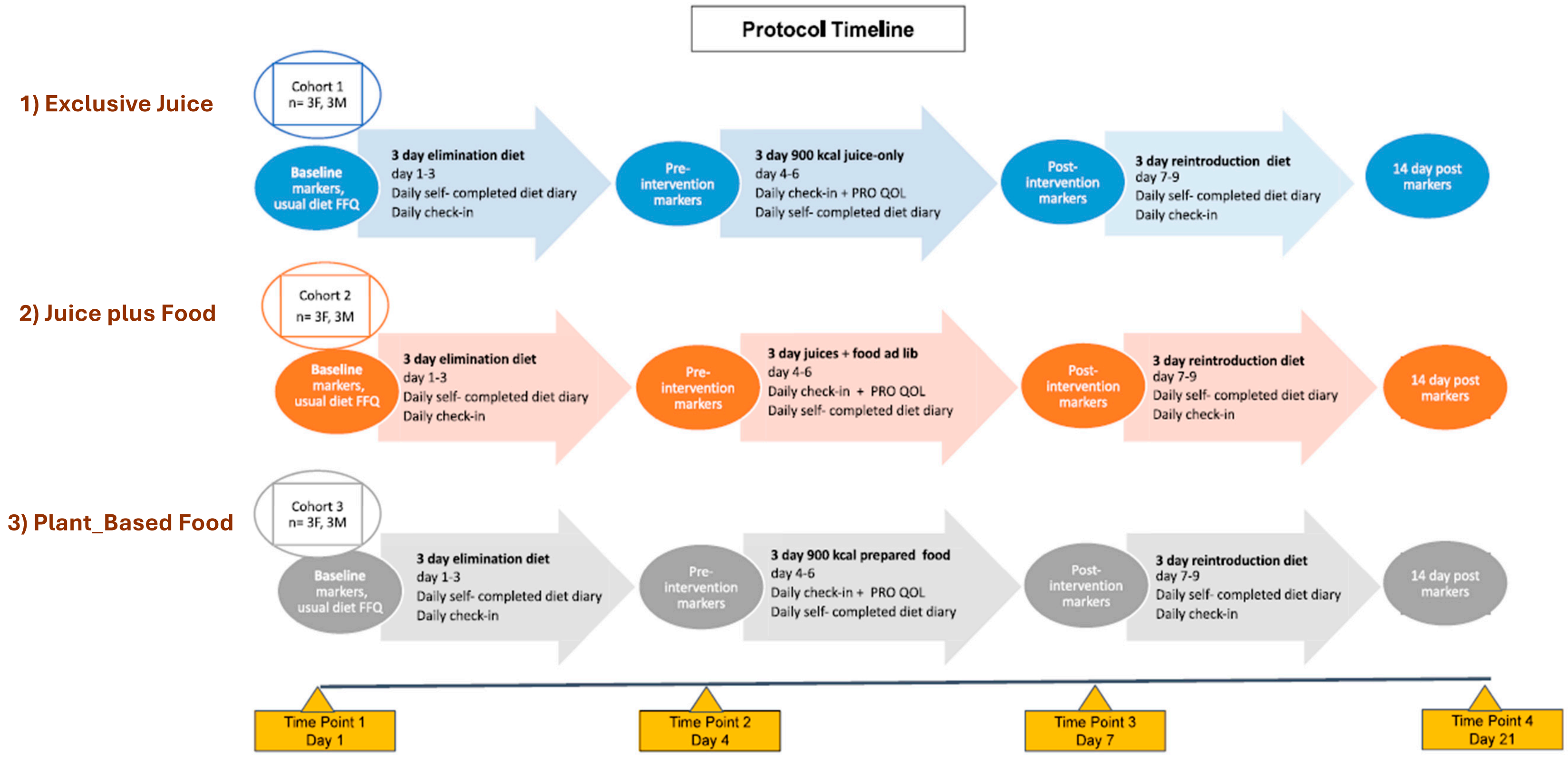

Table S1 Diet intervention summary.

**Nutrition Facts**	**Juice-Pac Shakur**		**Whitney Juice-ton**		**Juice Springsteen**		**Juice Bigalow**		**Lucky Juice-Iano**	
Quantity	1		1		2		1		1	
**Amount Per**	**16 oz**	**%RDA**	**16 oz**	**%RDA**	**16 oz**	**%RDA**	**16 oz**	**%RDA**	**16 oz**	**%RDA**
Calories	200		120		120		220		108	
Sodium (mg)	50		200		240		60		52	
Total Carbohydrates (g)	42		24		42		56		29	
Dietary Fiber (g)	2		2		4		0		5	
Sugars (g)	22		10		16		38		17	
Protein (g)	8		2		10		2		2	
Vitamin A		140%		240%		240%		0%		16%
Vitamin C		560%		100%		480%		8%		36%
Potassium (mg)	0		0		0		0		398	
Calcium		40%		4%		40%		0%		4%
Iron		30%		0%		40%		0%		6%
Ingredients	Green Apple, Kale, Cucumber, Celery, Parsley, Romaine, Lemon		Spinach, Cucumber, Celery, Carrot, Apple, Lemon		Celery, Apple, Kale, Chard, Ginger, Dandelion, Parsley, Lemon		Apple, Beet, Carrot, Ginger, Lemon		Pear (*when seasonally available: apple), Cucumber, Spinach, Lemon	

Table S2 Nutritional composition of the juices consumed as part of the exclusive juice diet and the juice *plus* food interventions.

Nutrition Facts	Loaded Cauliflower Mash (No Bacon)		Strawberry Cashew Yogurt		Power Green Veggie Quinoa Bowl		Herbed Root Vegetable Side		Spice Roasted Sweet Potato Side		Paleo Blueberry Muffin		Broccolini		Tomato and Roasted Garlic Soup	
**Amount Per**	**141.7g**	**%RDA**	**198.4g**	**%RDA**	**326.0g**	**%RDA**	**226.8g**	**%RDA**	**226.8g**	**%RDA**	**62.0g**	**%RDA**	**226.8g**	**%RDA**	**453.6g**	**%RDA**
Calories	75		453		353		222		229		162		81		187	
Calories from fat	25		292		188		34		20		106		8		57	
Total Fat (g)	2.5		32.4		20.9		3.7		2.2		11.7		0.9		6.3	
Saturated Fat (g)	0.2		6.6		2.9		0.4		1.6		3.3		0.1		1.1	
Trans Fat (g)	0		0		0		0		0		0		0		0	
Polyunsaturated Fat (g)	0		0		0.1		0.4		0		0		0.1		0	
Monounsaturated Fat (g)	0		0		0		2.6		0		0		0		0	
Cholesterol (mg)	0		0		0		0		0		36.4		0		0	
Sodium (mg)	130.7		81.8		545		224.6		349.7		87.8		149.9		278.3	
Total Carbohydrates (g)	12.3		33.1		35.3		45.6		66.1		13.2		16		27	
Dietary Fiber (g)	4.9		5.1		7.4		10.3		10.4		3.5		6.1		4.4	
Sugars (g)	4.2		14.8		4.2		13.2		13.7		6.9		3.9		13.8	
Added Sugars (g)	0		7.3		0		0		0		3.4		0		0	
Sugar Alcohol (g)	0		0		0		0		0		0		0		0	
Protein (g)	4.7		12.9		10.1		4.6		5.3		4.8		6.6		5.9	
Vitamin A		9.8%		0.10%		106.50%		306.60%		929.90%		1.60%		29.40%		114.20%
Vitamin C		140.7%		38.0%		166.30%		83.80%		13.00%		3.70%		338.90%		135.90%
Vitamin D		0%		0%		0%		0.00%		0.00%		2.00%		0.00%		0.00%
Vitamin E		15.7%		15.9%		28.90%		16.20%		7.60%		52.20%		9.00%		4.00%
Vitamin K		155.9%		21.6%		524.40%		85.70%		9.70%		0.10%		289.10%		25.50%
Vitamin B1 (thiamine)		17.3%		19.2%		17.80%		16.90%		17.10%		0.60%		11.00%		5.60%
Vitamin B2 (riboflavin)		15.3%		8.6%		18.40%		9.20%		12.60%		1.70%		15.90%		2.50%

Vitamin B3 (niacin)		8.2%		5.9%		8.60%		14.80%		10.10%		0.80%		7.40%		2.30%
Pantothenic acid		10.8%		5.5%		5.80%		12.40%		26.10%		0.40%		13.20%		2.50%
Vitamin B6		19.9%		14.3%		23.50%		22.30%		35.70%		2.40%		21.10%		12.20%
Vitamin B12		3.8%		0%		0%		0.00%		0.00%		1.50%		0.00%		0.00%
Folate		33.4%		8.3%		41.90%		27.80%		9.00%		1.90%		35.80%		4.30%
Calcium		7.1%		5.6%		19.70%		10.30%		10.30%		5.40%		11.20%		9.50%
Phosphorus		8.7%		34.7%		38.30%		17.80%		15.80%		3.10%		15.30%		8.70%
Potassium (mg)	601.1		520.4		789.4		1231.6		1126.7		101.8		734.2		693.6	
Magnesium		8.9%		43.9%		32.60%		17.20%		20.90%		4.30%		12.20%		9.80%
Zinc		4.1%		21.7%		13.90%		8.80%		7.00%		1.60%		6.40%		5.30%
Selenium		1.8%		17.3%		8.70%		4.00%		3.30%		0.20%		8.50%		4.30%
Copper		5.2%		60.7%		39.80%		17.80%		25.40%		1.40%		6.10%		14.10%
Manganese		17.0%		59.9%		67.80%		46.40%		43.30%		11.50%		27.90%		22.80%
Ingredients	Cauliflower, scallions, almond, nutritional yeast, parsley, thyme, rosemary, garlic, salt, black pepper		Yogurt, cashew, raw honey, lemon, coconut water, vanilla extract, psyllium, salt, strawberry, lemon, honey, salt, granola, almonds, sunflower seeds, pecans, cashews, almond flour, maple syrup, clover honey, coconut oil, flax seed, vanilla extract, cinnamon, sea salt		Quinoa, arugula, broccoli, asparagus, kale, mustard greens, parsley, salt, sauce verte, basil, arugula, evo, garlic, red onion, lemon, capers, dijon, red fresh pepper, salt		Parsnip, carrot, turnip, redskin potato, high-oleic sunflower oil, parsley, salt, black pepper		Sweet potato, chili powder, paprika, coconut oil, salt		Almond flour, coconut flour, banana, almond butter, almonds, baking powder, salt, baking soda, vanilla extract, coconut oil, maple syrup, coconut milk, pastured eggs, cinnamon, blueberries		Broccolini, garlic, salt, black pepper, lemon zest, chili flake		Soup tomato, vegetable broth, onion, red bell pepper, garlic, carrot, cashew, tomato paste, olive oil, basil, salt, black pepper, chili flakes, garnish basil	

Table S3 List of the food products used for the plant-based, whole food intervention.

Factor - Sex	Df	SumsOfSqs	F.Model	R2	p.value	p.adjusted	sig
**Cheek Unweighted UniFrac**							
1-baseline-Female vs 1-baseline-Male	1	0.184	0.971	0.075	0.425	1	-
2-pre-intervention-Female vs 2-pre-intervention-Male	1	0.198	1.025	0.079	0.401	1	-
3-Food_Plant_based-Female vs 3-Food_Plant_based-Male	1	0.166	0.928	0.236	0.700	1	-
3-Food+Juice-Female vs 3-Food+Juice-Male	1	0.142	0.474	0.192	1.000	1	-
3-Juice-Female vs 3-Juice-Male	1	0.173	0.879	0.305	1.000	1	-
4-Food_Plant_based-Female vs 4-Food_Plant_based-Male	1	0.234	1.455	0.327	0.200	1	-
4-Food+Juice-Female vs 4-Food+Juice-Male	1	0.356	3.097	0.608	0.333	1	-
4-Juice-Female vs 4-Juice-Male	1	0.178	0.828	0.293	0.750	1	-
**Cheek Weighted UniFrac**							
1-baseline-Female vs 1-baseline-Male	1	0.030	0.612	0.048	0.832	1	-
2-pre-intervention-Female vs 2-pre-intervention-Male	1	0.035	0.878	0.068	0.531	1	-
3-Food_Plant_based-Female vs 3-Food_Plant_based-Male	1	0.069	0.905	0.232	0.400	1	-
3-Food+Juice-Female vs 3-Food+Juice-Male	1	0.042	0.332	0.142	1.000	1	-
3-Juice-Female vs 3-Juice-Male	1	0.014	0.608	0.233	1.000	1	-
4-Food_Plant_based-Female vs 4-Food_Plant_based-Male	1	0.028	0.991	0.248	0.400	1	-
4-Food+Juice-Female vs 4-Food+Juice-Male	1	0.020	0.646	0.244	0.667	1	-
4-Juice-Female vs 4-Juice-Male	1	0.015	0.500	0.200	1.000	1	-
**Saliva Unweighted UniFrac**							
1-baseline_Female vs 1-baseline_Male	1	0.130	0.882	0.068	0.695	1	-
2-pre-intervention_Female vs 2-pre-intervention_Male	1	0.139	0.914	0.071	0.675	1	-
3-Food_Plant_based_Female vs 3-Food_Plant_based_Male	1	0.190	1.094	0.267	0.300	1	-
3-Food+Juice_Female vs 3-Food+Juice_Male	1	0.170	1.160	0.367	0.333	1	-
3-Juice_Female vs 3-Juice_Male	1	0.142	1.103	0.269	0.400	1	-
4-Food_Plant_based_Female vs 4-Food_Plant_based_Male	1	0.195	1.109	0.270	0.200	1	-
4-Food+Juice_Female vs 4-Food+Juice_Male	1	0.122	0.797	0.285	1.000	1	-
4-Juice_Female vs 4-Juice_Male	1	0.132	0.806	0.212	0.800	1	-
**Saliva Weighted UniFrac**							
1-baseline_Female vs 1-baseline_Male	1	0.027	0.760	0.060	0.568	1	-
2-pre-intervention_Female vs 2-pre-intervention_Male	1	0.016	0.356	0.029	0.782	1	-
3-Food_Plant_based_Female vs 3-Food_Plant_based_Male	1	0.015	0.235	0.073	0.700	1	-
3-Food+Juice_Female vs 3-Food+Juice_Male	1	0.071	2.785	0.582	0.333	1	-
3-Juice_Female vs 3-Juice_Male	1	0.155	3.671	0.550	0.100	1	-
4-Food_Plant_based_Female vs 4-Food_Plant_based_Male	1	0.014	0.648	0.178	0.900	1	-
4-Food+Juice_Female vs 4-Food+Juice_Male	1	0.015	0.464	0.188	1.000	1	-
4-Juice_Female vs 4-Juice_Male	1	0.020	0.316	0.095	0.600	1	-
**Fecal Unweighted UniFrac**							
1-baseline-Female vs 1-baseline-Male	1	0.280	1.211	0.131	0.165	1	-
2-pre-intervention-Female vs 2-pre-intervention-Male	1	0.248	0.988	0.165	0.514	1	-
3-Food_Plant_based-Female vs 3-Food_Plant_based-Male	1	0.223	1.005	0.334	0.667	1	-
3-Food+Juice-Female vs 3-Food+Juice-Male	1	0.290	1.396	0.411	0.333	1	-
3-Juice-Female vs 3-Juice-Male	1	0.222	0.926	0.236	0.700	1	-
4-Food_Plant_based-Female vs 4-Food_Plant_based-Male	1	0.299	1.266	0.297	0.100	1	-
4-Food+Juice-Female vs 4-Food+Juice-Male	1	0.200	0.739	0.425	1.000	1	-
4-Food+Juice-Male vs 4-Juice-Female	1	0.171	0.702	0.190	1.000	1	-
**Fecal Weighted UniFrac**							
1-baseline-Female vs 1-baseline-Male	1	0.184	2.529	0.240	0.060	1	-
2-pre-intervention-Female vs 2-pre-intervention-Male	1	0.068	0.720	0.126	0.800	1	-
3-Food_Plant_based-Female vs 3-Food_Plant_based-Male	1	0.016	0.594	0.229	0.667	1	-
3-Food+Juice-Female vs 3-Food+Juice-Male	1	0.030	0.677	0.253	0.667	1	-

3-Juice-Female vs 3-Juice-Male	1	0.048	0.715	0.192	0.900	1	-
4-Food_Plant_based-Female vs 4-Food_Plant_based-Male	1	0.090	1.201	0.286	0.300	1	-
4-Food+Juice-Female vs 4-Food+Juice-Male	1	0.070	0.715	0.417	0.667	1	-
4-Food+Juice-Female vs 4-Juice-Female	1	0.033	0.644	0.244	0.750	1	-

Table S4 Pairwise Comparisons analysis based on sex factor and diet interventions using weighted UniFrac and unweighted UniFrac distances across the three body sites: cheek, saliva, and fecal samples.

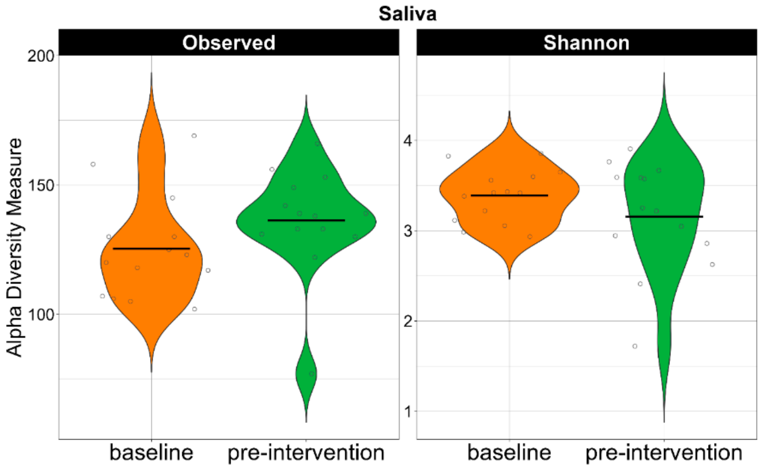

Figure S1 Observed ASVs and Shannon alpha diversity violin plots in cheek samples at baseline and pre-intervention time points in response to the elimination diet.

F

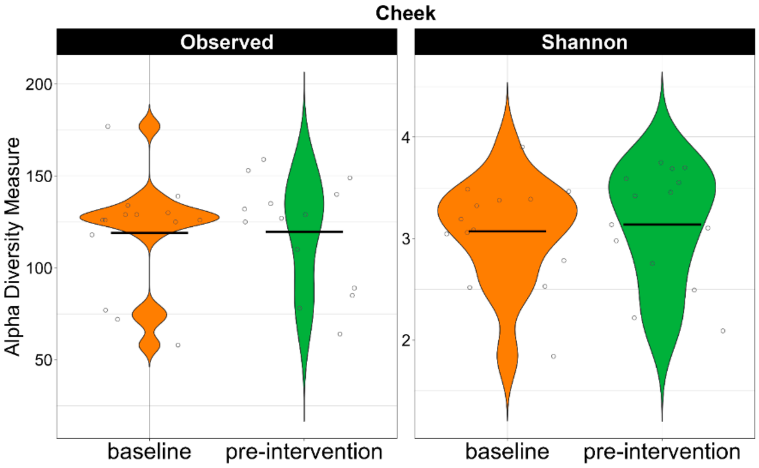

Figure S2 Observed ASVs and Shannon alpha diversity violin plots in saliva samples at baseline and pre-intervention time points in response to the elimination diet.

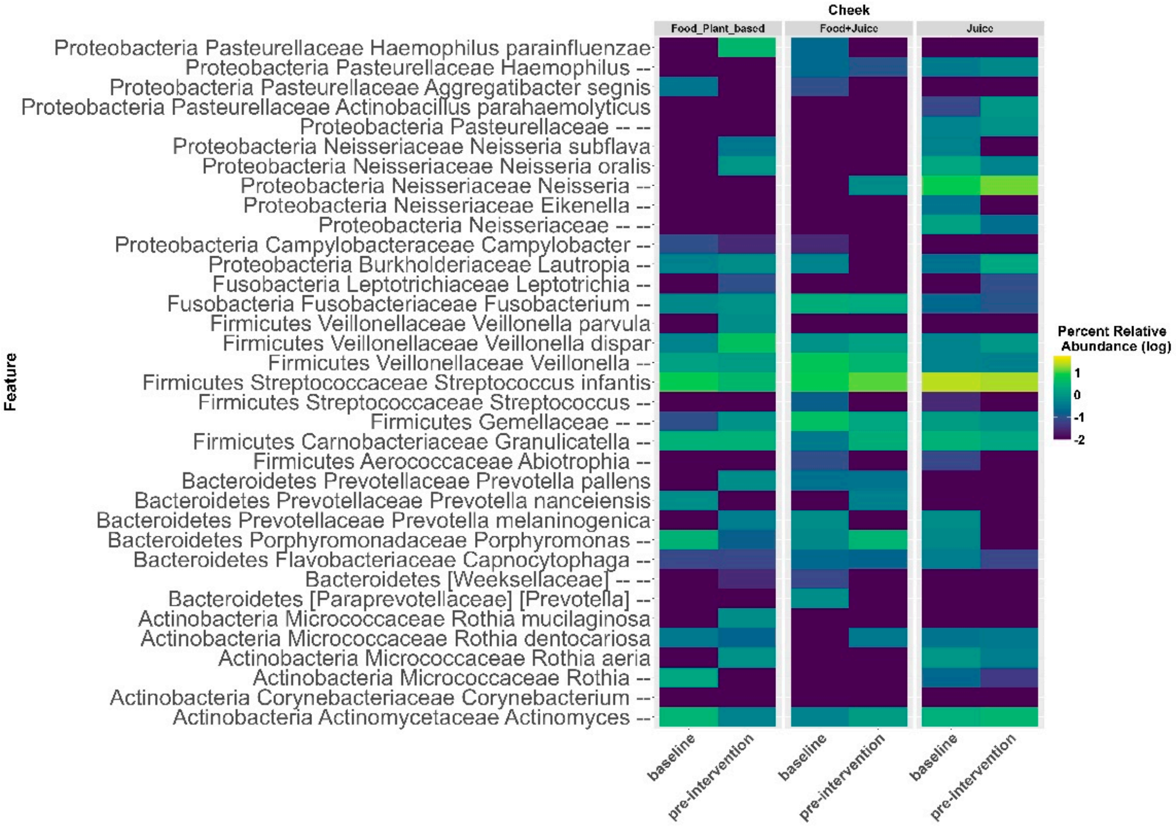

Figure S3 Heat map comparison of the relative taxa abundance in cheek samples from participants at baseline point and pre-intervention.

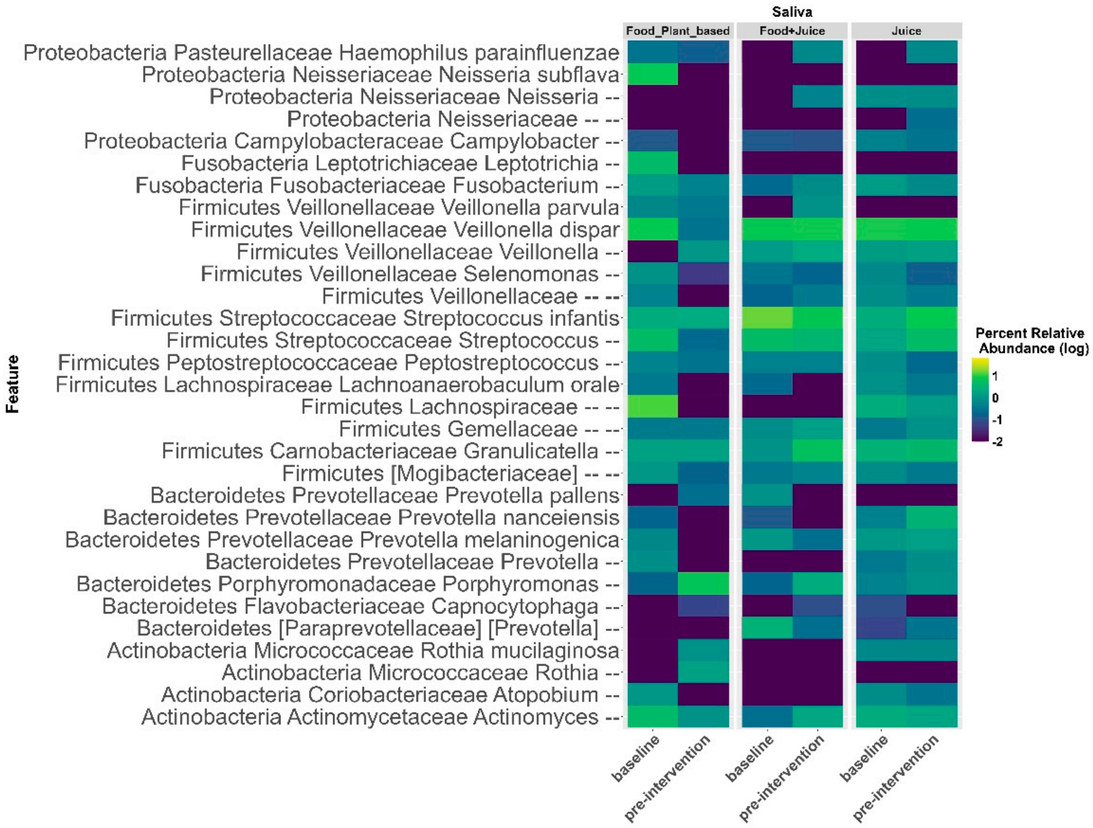

Figure S4 Relative abundance heatmap of genus and bacterial species in saliva samples from baseline and pre-intervention intervention time points, in response to elimination diet, reported as log10 of percent abundance.

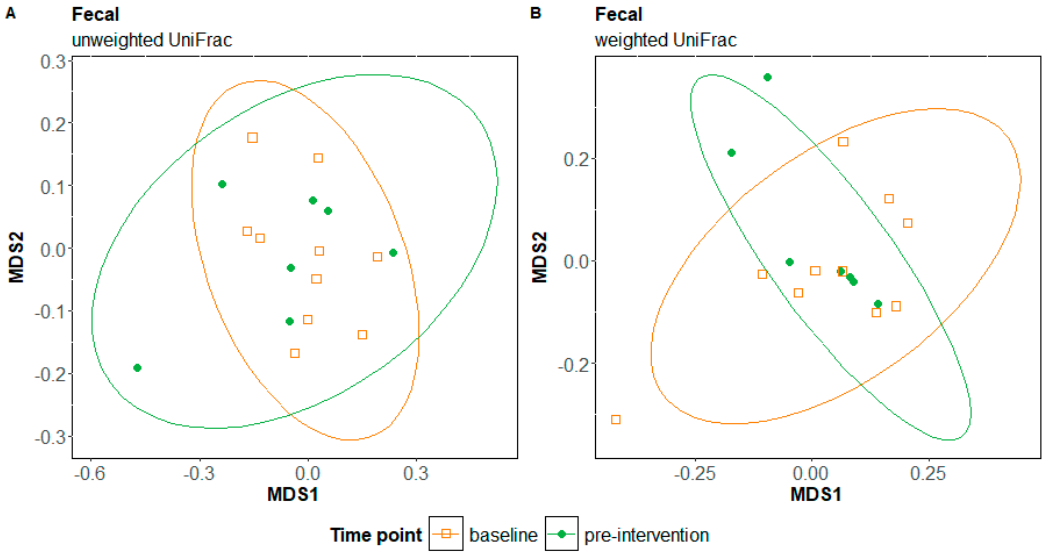

Figure S5 Non-metric multidimensional scaling plots (MDS) with the Unweighted Unifrac (A) and Weighted Unifrac distance (B) on fecal samples at baseline and pre-intervention time point, in response to elimination diet.

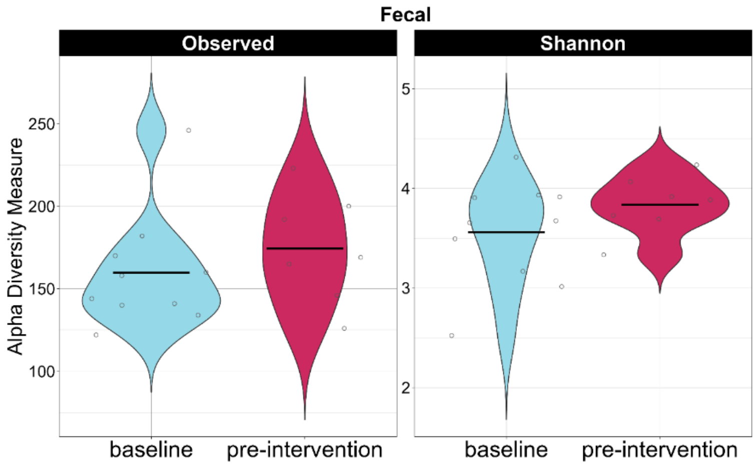

Figure S6 Observed ASVs and Shannon alpha diversity violin plots in fecal samples at baseline and pre-intervention time points, in response to the elimination diet.

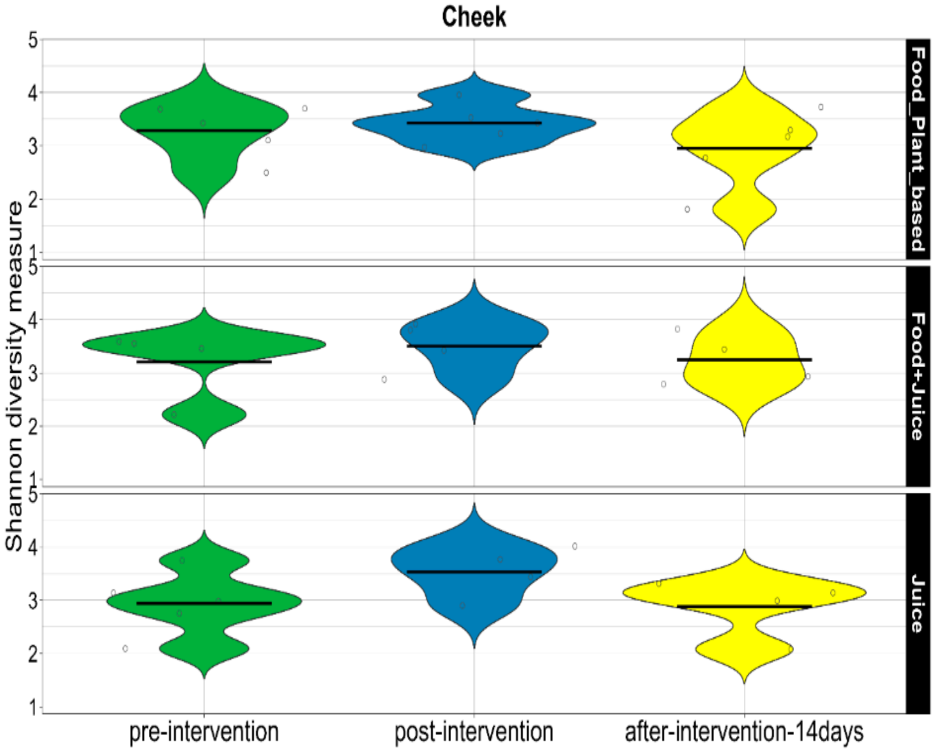

Figure S7 Shannon diversity violin plots in cheek samples at pre-intervention, post-intervention and 14 days post-intervention time points for the three diet types.

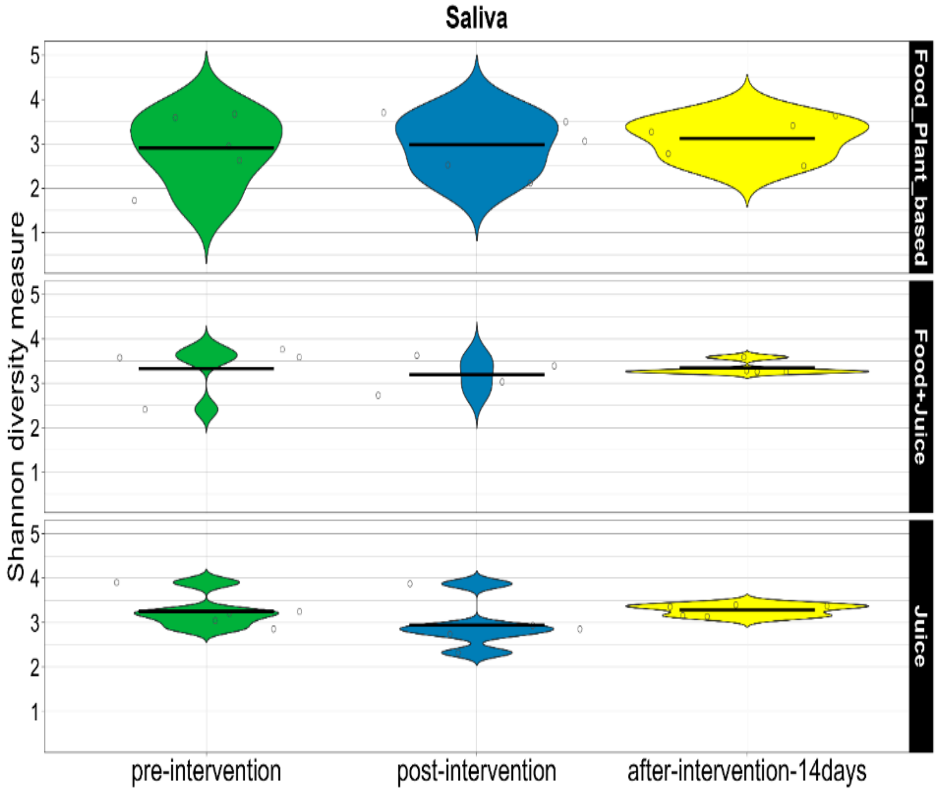

Figure S8 Shannon diversity violin plots in saliva samples at pre-intervention, post-intervention and 14 days post-intervention time points for the three diet types.

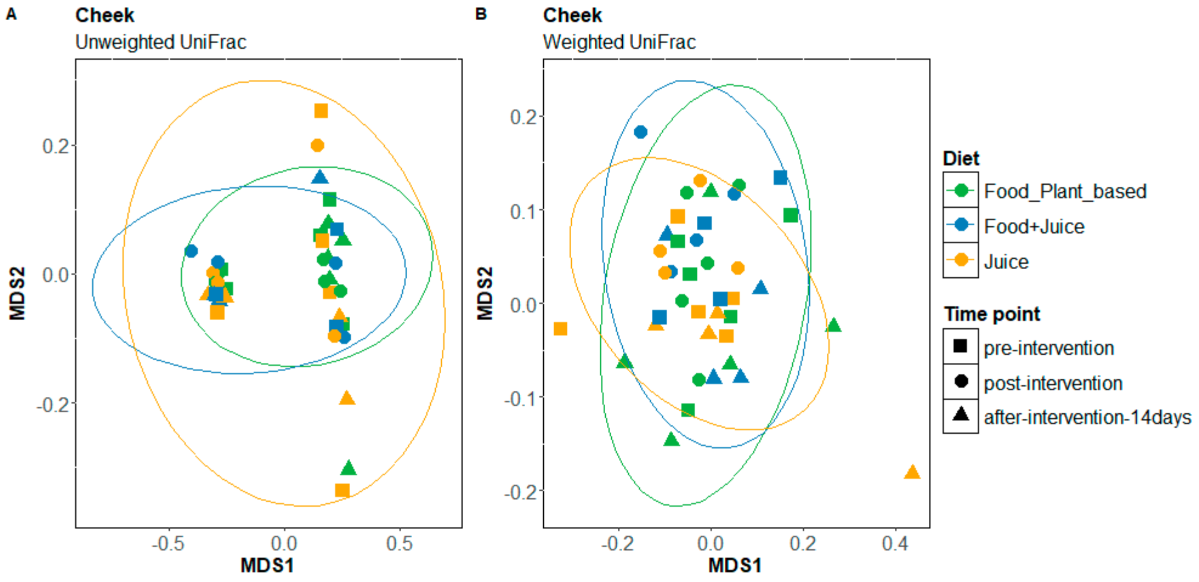

Figure S9 Multidimensional scaling plots (MDS) on Unweighted Unifrac (A) and Weighted Unifrac distance matrix (B) performed on cheek samples at pre-intervention, post-intervention and after-intervention-14days.

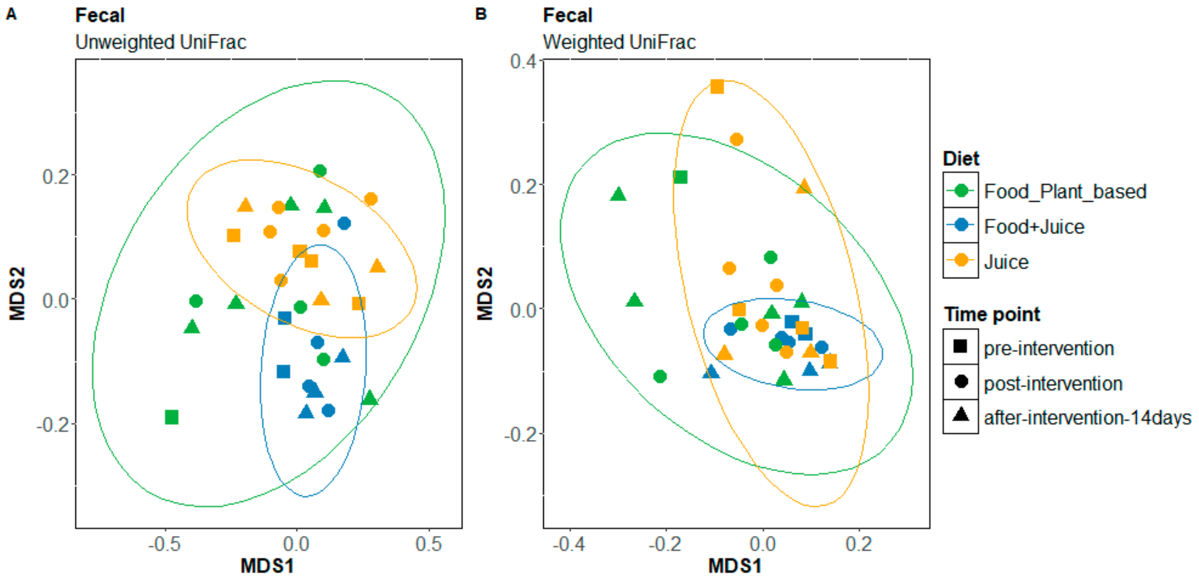

Figure S10 Multidimensional scaling plots (MDS) on Unweighted Unifrac (A) and Weighted Unifrac distance matrix (B) performed on fecal samples at pre-intervention, post-intervention and after-intervention-14days.

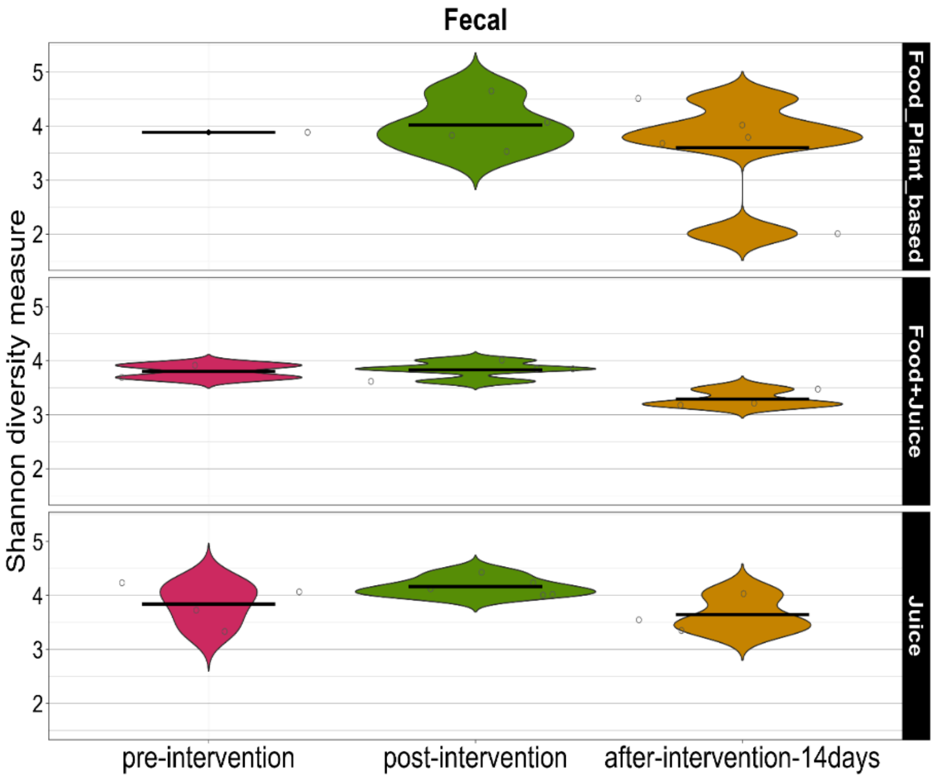

Figure S11 Shannon diversity violin plots in fecal samples at pre-intervention, post-intervention and 14 days postr-intervention time points for the three diet types.

A)

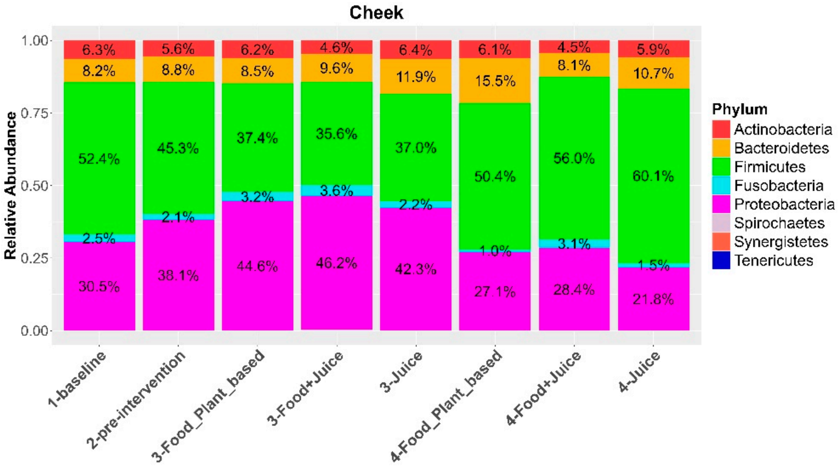

B)

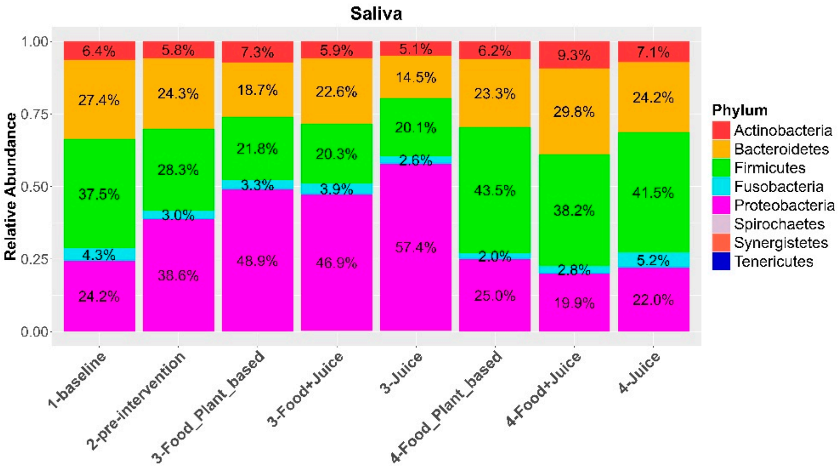

C)

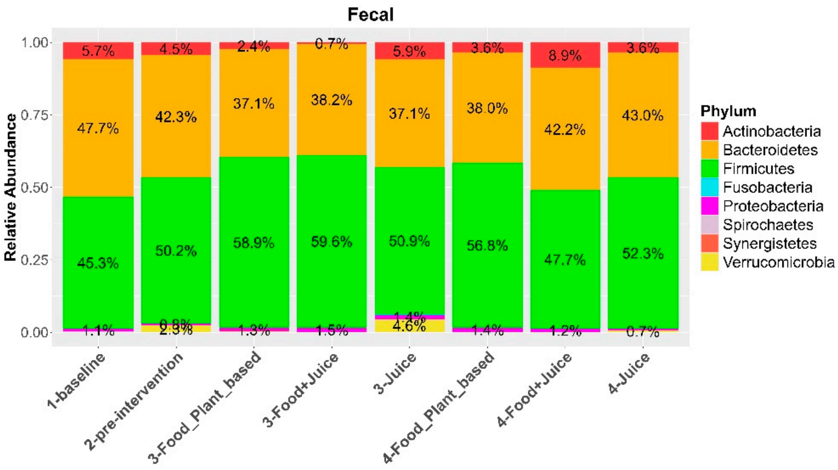

Figure S12 Relative abundance of bacterial phyla in A) Cheek, B) saliva, and C) fecal samples at baseline (1-baseline), pre-intervention (2-pre-intervention), post-intervention (3-Food_Plant_based, 3-Food+Juice, 3-Juice), 14 days post intervention (4-Food_Plant_based, 4-Food+Juice, 4-Juice) time points.
